# Modeling the 2022 Mpox Outbreak with a Mechanistic Network Model

**Published:** 2025-05-08

**Authors:** Emma G. Crenshaw, Jukka-Pekka Onnela

**Affiliations:** 1 Department of Biostatistics, Harvard TH Chan School of Public Health, 677 Huntington Avenue, Boston, 02115, MA, USA.

**Keywords:** Mpox, MSM, network modeling, simulation

## Abstract

**Background:**

The 2022 outbreak of mpox affected more than 80,000 individuals worldwide, most of whom were men who have sex with men (MSM) who likely contracted the disease through close contact during sex. Given the unprecedented number of mpox infections and the new route of infection, there was substantial uncertainty about how best to manage the outbreak.

**Methods:**

We implemented a dynamic agent-based network model to simulate the spread of mpox in a United States-based MSM population. This model allowed us to implement data-informed dynamic network evolution to simulate realistic disease spreading and behavioral adaptations.

**Results:**

We found that behavior change, the reduction in one-time partnerships, and widespread vaccination are effective in preventing the transmission of mpox and that earlier intervention has a greater effect, even when only a high-risk portion of the population participates. With no intervention, 16% of the population was infected (25th percentile, 75th percentiles of simulations: 15.3%, 16.6%). With vaccination and behavior change in only the 25% of individuals most likely to have a one-time partner, cumulative infections were reduced by 30%, or a total reduction in nearly 500 infections (mean: 11.3%, $P_{25\%}$ and $P_{75\%}$: 9.6%, 13.5%). Earlier intervention further reduces cumulative infections; beginning vaccination a year before the outbreak results in only 5.5% of men being infected, averting 950 infections or nearly 10% of the total population in our model. We also show that sustained partnerships drive the early outbreak, while one-time partnerships drive transmission after the first initial weeks. The median effective reproductive number, $R_{*}^{t}$, at t=0 days is 1.30 for casual partnerships, 1.00 for main, and 0.6 for one-time. By t=28, the median $R_{*}^{t}$ for one-time partnerships has more than doubled to 1.48, while it decreased for casual and main partnerships: 0.46 and 0.29, respectively.

**Conclusion:**

With the ability to model individuals’ behavior, mechanistic networks are particularly well suited to studying sexually transmitted infections, the spread and control of which are often governed by individual-level action. Our results contribute valuable insights into the role of different interventions and relationship types in mpox transmission dynamics.

## 1 Background

Mpox is a vaccine-preventable viral infection that, before 2022, was primarily zoonotic and restricted to endemic areas of central and western Africa[1, 2]. However, in 2022, there was a significant global outbreak. Within a year, more than 87,000 cases were identified, approximately 30,000 of which were in the United States[3]. These cases disproportionately affected gay, bisexual, and other men who have sex with men (MSM)[3]. This outbreak marked a significant shift in transmission dynamics, with human-to-human transmission driving the epidemic. Transmission occurred primarily through contact with infectious lesions, with most cases being associated with sexual contact[1, 4, 5].

Due to the change in transmission modality from zoonotic to person-to-person, there was a great deal of clinical uncertainty about the transmissibility of the disease and uncertainty about how best to prevent future outbreaks. The concentration of cases among MSM highlighted the importance of understanding mpox spread in sexual networks. There are two general frameworks of network modeling, statistical and mechanistic. Within each of these, the models can be either static or dynamic. Mechanistic network models, such as agent-based models, have been used previously in the study of HIV/AIDS and other sexually transmitted infections (STIs) in this population. These models are able to represent important aspects of sexual networks, such as repeated interactions with partners which cannot be captured without a network framework, and they can directly represent an individual’s behavioral mechanisms, such as partnership formation, concurrency, or treatment and vaccination seeking[6–10]. Thus, they are particularly well-suited to characterizing disease transmission governed by individual-level action, such as sexual contact patterns and partnership dynamics.

Recent modeling studies have focused on ascertaining epidemiological and clinical features of the disease, such as the transmission probability per sexual contact [2, 11, 12], the incubation period [12–14], and the basic reproductive number $\left(R_{0}\right)$ [10, 12, 15]. Focusing on the 2022 outbreak, there have also been studies looking at the relative importance of different interventions in ending the outbreak in the US and abroad[11, 12, 15–18]. However, a paucity of literature is available that synthesizes lessons learned from the 2022 outbreak and explores the range of outcomes with alternative interventions or intervention timings. Additionally, current network modeling approaches in the literature focus on either statistical network modeling using exponential random graphs[5, 15, 17, 19] or do not incorporate dynamic sexual partnerships[10].

In this paper, we outline how we integrated data about the clinical course of mpox, sexual network characteristics of MSM, and individual- and policy-level interventions to generate a model of mpox transmission in a population of MSM in the United States. We also describe several simulations that compare the impact of the timing of interventions at different levels of compliance and intervention access to showcase the usefulness of these models as a way to inform individual behavior and health policy when the clinical features of an emerging outbreak are still uncertain.

## 2 Methods

### 2.1 Network Characterization

We developed an agent-based model to simulate a dynamic sexual network of 10,000 MSM. The network is initialized as a configuration model with a degree distribution that is consistent with real-world sexual networks of MSM (Table 1)[6, 20–22]. While the standard configuration model is limited to static networks, temporal variants of the model have also been recently proposed, such as in Le (2024)[6, 20]. Our extension of the standard configuration model has two primary changes - we consider three different types of network ties (main, casual, and one-time partnerships), and we allow the graph to evolve over time to better represent the dynamic nature of sexual relationships. We chose network parameters to match empirical network structures reported in observational studies of MSM in the United States [21, 23]. Primarily, we use empirically defined population-level proportions of individuals in different relationship types, defined as the number of main and casual partners an individual will have concurrently, and population-level heterogeneity in the daily probability that an individual will have one-time contact. The network does not assume assortativity based on node features, such as preferred relationship type or sexual activity risk group; however, nodes seeking more partnerships are more likely to partner with other high-degree nodes because they are over-represented in the pool of potential partners seeking relationships.

**Fig. 1:**
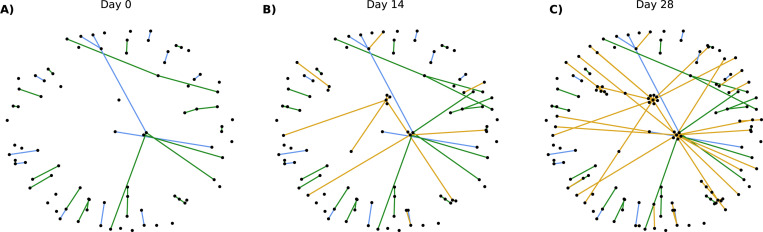
Cumulative edges for an ego-centric sample of 100 nodes and their alters. Graphs show for day 0 (panel A), day 14 (Panel B), and day 28 (Panel C). Main partnerships are shown in blue, casual partnerships in green, and one-time partnerships in yellow. Nodes selected are treated as ‘egos’ and their partners as ‘alters’. Thus, the only edges shown are those that involve an ego node. The number of main, casual, and one-time partnerships are 12, 23, 0 (Panel A); 12, 27, 15 (Panel B); 12, 27, 54 (Panel C)

To demonstrate how the simulated sexual networks evolve over time, we show snapshots of the cumulative edges for an ego-centric sample of 100 nodes formed over 28 days (Figure 1). In these network visualizations, the 100 nodes sampled uniformly at random are treated as egos, and their partners (alters) are added to the visualization if they form an edge with an ego. In addition to ego-ego ties, the ego-alter ties are shown. From these visualizations, the importance of one-time partnerships in connecting different parts of the network over time becomes apparent.

#### 2.1.1 Main and casual partnerships

The numbers of main and casual partnerships in the network follow the proportions in Table 1, based on estimates from a national survey of MSM distributed between 2017 and 2019[21], supplemented by data from two Atlanta-based surveys[23]. Each node is assigned probabilistically to a combination of main/casual partnership counts so that the final population percentages are matched to the real-world estimates and maintains that throughout the simulation. The probability of sexual contact on a given day with a particular partner is defined as 22% for main partners and 14% for casual partners, as found in the empirical surveys[21, 23]. The duration of each main or casual partnership is randomly defined via a draw from a geometric distribution parameterized by the mean partnership duration from observational studies: 407 days for main partnerships and 166 for casual partnerships[7, 21]. Once a given partnership has reached its randomly pre-determined duration, the edge is dissolved, and nodes are randomly rewired to other nodes with the same type of partnership stub (”half edge”) available. Nodes cannot rewire to their most recent connection; if no other nodes are available, they will wait to form another edge until another partnership of the desired type is dissolved to create new connections to available partners. In the appendix, we have characterized the typical wait time for a node when rewiring. For a network of size N=10,000, fewer than 1% of nodes looking to form a new main partnership are not able to do so immediately, and fewer than 0.002% of those looking to form a casual partnership are not able to do so immediately (Table A1). A more detailed description of the network structure and summaries of network statistics are also available in the appendix.

#### 2.1.2 One-time partnerships

Nodes were also randomly assigned to a stratum of sexual activity, defined as their daily probability of forming a one-time partnership, which is constant throughout the simulation. These probabilities are based on the same studies as the parameters for main and casual partnerships[21, 23]. Each node has a 19% probability of being assigned to strata 1–5 and a 5% probability of being assigned to strata 6; in this way, the strata can be thought of as quintiles of risk of one-time partnership with the addition of a ‘high-risk’ group. This assumption is consistent with other modeling studies and is based on surveys of MSM[7, 15, 19, 24]. These probabilities range from 0, when an individual will never have a one-time partner, to 0.286 in the high-risk group, which corresponds to approximately 8 one-time partners per month on average. By definition, the duration of a one-time partnership is one time step, and the probability of sexual contact is one.

### 2.2 Epidemic Parameters

To model the spread of mpox on this network, we used a discrete-time stochastic susceptible-exposed-infected-recovered (SEIR) transmission model. Each day, the occurrence of sexual contact in an existing partnership is treated as an independent random event. The probability of disease transmission in serodiscordant pairs is defined as the product of the probability of sexual contact and the overall transmission probability. Infected individuals are randomly assigned a given amount of time that they will be in the exposed and infected states, according to the distributions in Table 1. Mean values for the distributions were defined as the average estimated time for each state in accordance with existing literature[1, 2, 13, 25–27]. Once an individual’s time in the current state has expired, they move to the next state. Initially, 0.1% of nodes are infected at time t=0. These nodes were selected randomly from the 25% of nodes most likely to have a one-time sexual partnership, i.e., those in the top two strata of sexual activity, to ensure that it is unlikely the spreading process dies out by chance within the first few time steps.

During the 2022 outbreak, individuals were recommended to abstain from sexual contact until resolution of their symptoms. However, the severity of symptoms ranged, and it is possible that people may not have recognized that they were infected, particularly early in the syndromic stage, delaying isolation. This is especially true early in the outbreak, as people were less likely to be aware of the symptoms of mpox. To account for this uncertainty in our simulations, we assumed that 80% of individuals would seek clinical care, allowing for heterogeneity in behavior, symptom severity, and access to healthcare. Once an individual was infected, we assumed a 15-day diagnosis delay at the outbreak’s start[17]. This delay decreased by approximately one day every four days of the simulation to simulate increased awareness by individuals and healthcare providers as the outbreak progressed[28]. The minimum allowed delay was 5 days. Once diagnosed, the node is assumed to completely isolate from all partners. A sensitivity analysis in the supplement shows a less optimistic isolation scheme; instead of full isolation, nodes that isolate will avoid one-time partnerships but only decrease their probability of contact with main and casual partners by 50%.

### 2.3 Interventions

We investigated several potential interventions at different levels of intensity to determine their effect on cumulative mpox incidence after 250 days. Based on the literature and reports of real-world interventions, we simulated individual behavior change and vaccination. Our main results are presented for vaccination, which begins at 30 days, and behavioral change, which begins at 70 days. These timings were selected to represent the real-world outbreak: the first mpox case was diagnosed on May 17, 2022, vaccines became available on May 22, 2022, and a survey of MSM reported that individuals were engaging in behavioral change was conducted on August 5–15, 2022[29, 30]. Assuming that the virus was circulating two weeks shortly before the first case was diagnosed, the 30-day and 70-day timings reflect these real-world interventions. Parameters used in simulation are in Table 1.

#### 2.3.1 Behavior change

According to preliminary survey results, approximately 50% of MSM in the US reduced their overall number of sexual partners, and 50% reduced their number of one-time partners[16]. We wanted to examine the results if all members of the network were to reduce their sexual activity compared to only those most likely to form a one-time partnership doing so. To investigate this, we simulated a 50% reduction in all individuals’ probability to form a one-time partnership compared to the same reduction in only sexual activity strata 5 and 6, the 25% of individuals with the greatest probability of forming a one-time partnership. For example, for individuals in stratum 6, this would correspond to a decrease from 8 one-time partners per month on average to 4 one-time partners.

#### 2.3.2 Vaccination

The vaccine approved in the US for the prevention of mpox, JYNNEOS, requires two doses 28 days apart[31]. Vaccine coverage is still relatively low; approximately 22.7% of MSM have received a first dose as of January 2023 [32]. In our simulation, we modeled the availability of vaccines using the Center for Disease Control and Prevention’s (CDC) report of the number of vaccinations administered per week in the US between May 22, 2022 and July 11, 2023. The average number of vaccines administered per day was adjusted for the population size of our network and used as the total number of vaccinations to administer each day of the simulation. Further details and the data used are available in the supplement.

Given that the vaccine supply was limited at the beginning of the outbreak, we simulated two vaccination schemes: random vaccination and vaccination of those in the highest stratum of sexual activity. Additionally, we looked at the effect of fast-tracking or delaying vaccine introduction to determine the sensitivity of disease transmission to potential improvements in epidemic preparedness.

### 2.4 Analyses

**Fig. 2:**
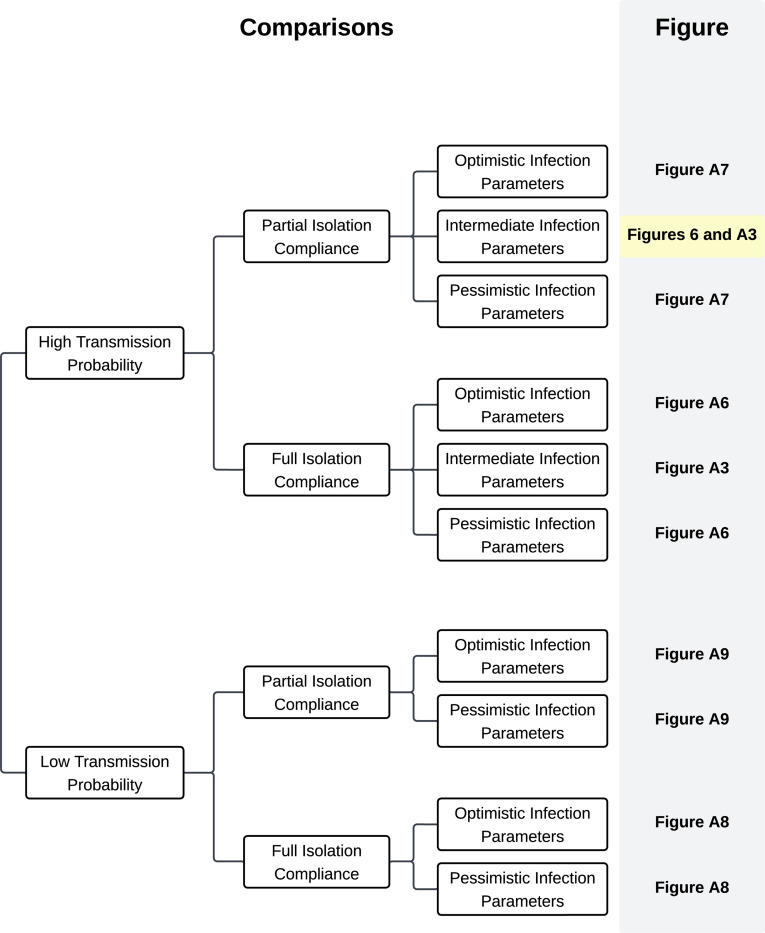
Explanation of Figures Comparisons of different intervention timings and intensities under different simulation conditions can be found in the indicated figures. The primary comparison results are in Figure 6 and copied in Figure A3 to enhance comparability.

All simulations and analyses were done in Python, and the code has been made publicly available[33]. Due to the stochastic nature of the model, wide variations in the simulations are possible. For example, it is possible that all infected individuals recover before infecting a partner, leading to the outbreak ending early. Similarly, given that the network is sparse, it is possible that the infection may become isolated within a particular portion of the network. Therefore, each analysis is the result of 100 independent simulations. Due to the complexity of a mechanistic network model, confidence intervals cannot be calculated; instead, we provide point-wise averages with the 25th and 75th percentiles of final cumulative infections to show the variability in simulation results. The effective reproductive number at time t for a particular simulation, $R_{*}^{t}$, is calculated as the average number of infections secondary to any node that was infectious in the week before time t. At time t=0, this is equivalent to $R_{0}$, the basic reproduction number. Results are presented as the average $R_{*}^{t}$ over the 100 simulations. Due to the number of sensitivity analyses, we included Figure 2 to show help readers navigate to figures showing the comparison of different intervention timings under different simulation conditions.

## 3 Results

We present the results of several analyses, comparing the cumulative number of infections across different intervention types and timings. Our primary results show behavioral change beginning at day 70 of the simulation and vaccination beginning at day 30 and assume that individuals who are diagnosed comply with full isolation. Sensitivity analyses examining the impact of partial isolation compliance and more or less optimistic estimates of the disease-spreading process can be found in the appendix.

### 3.1 Comparison of interventions

**Fig. 3:**
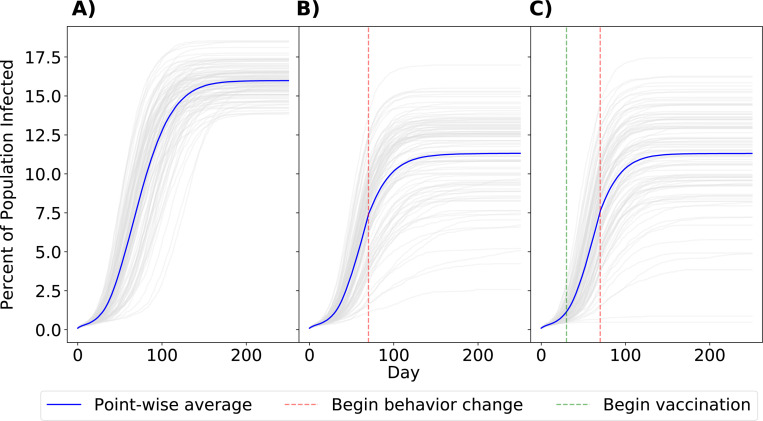
Comparison of universal interventions. Panels indicate the percent of network infected with mpox after 250 days with no intervention (Panel A), universal behavior change (Panel B), or universal behavior change with vaccination (Panel C). Grey lines denote individual simulations. The point-wise average is shown in blue. Vertical lines indicate the day of intervention initiation.

Our baseline model simulates an epidemic where no personal intervention or policy is in place to prevent the spread of mpox. In this scenario, we see a rapid increase in cases until more than 15% of the population of MSM is infected (mean percent of population infected: 15.98%, 25th and 75th percentiles of infections: 15.31%, 16.58%). To get a better understanding of the impact of behavior change and vaccination in preventing infection, we modeled universal behavior change, a 50% reduction in the probability of having a one-time partner, beginning at day 70 of our simulation and universal availability of vaccination beginning at day 30 (Figure 3). From behavior change alone, we see a 30% reduction in the total number of cases (mean: 11.32%, $P_{25\%}$ and $P_{75\%}$: 9.56%, 13.11%), translating to approximately 500 averted infections. Adding a universal availability of vaccination does not meaningfully affect the results (mean: 11.31% $P_{25\%}$ and $P_{75\%}$: 9.59%, 13.51%).

**Fig. 4:**
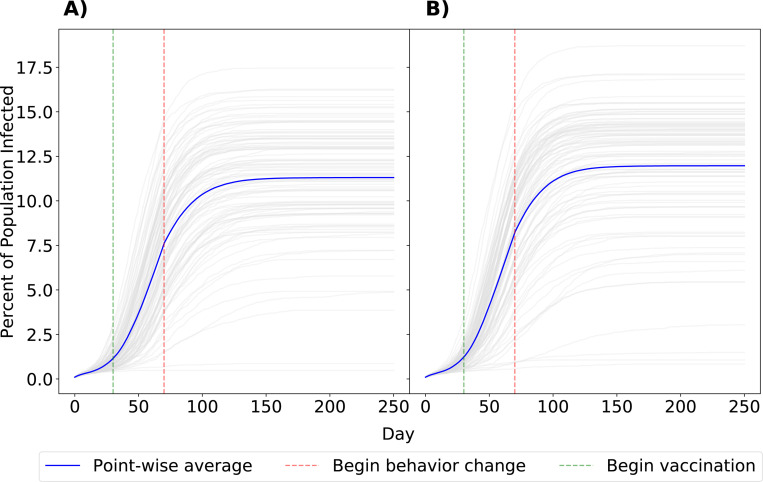
Comparison of scenarios with universal or targeted intervention. Panels indicate the percent of network infected with mpox after 250 days with universal behavior change with vaccination (Panel A) or intervention only in the 25% of men most likely to have a one-time partner (Panel B). Grey lines denote individual simulations. The point-wise average is shown in blue. Vertical lines indicate the day of intervention initiation.

**Fig. 5:**
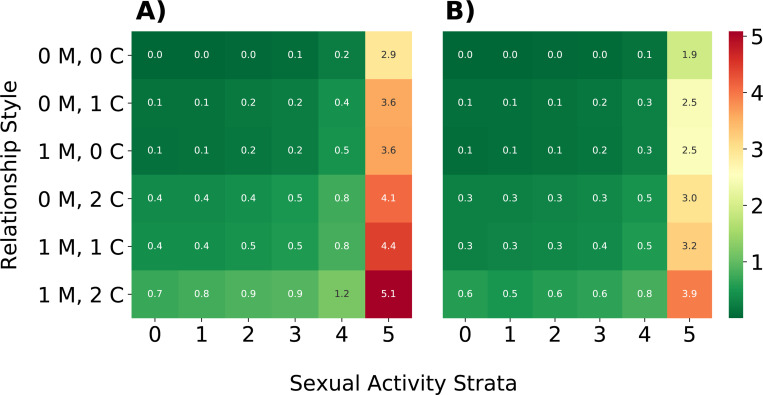
Comparison of average number of at-risk sexual interactions by relationship type and sexual activity strata Cell values indicate the average number of serodiscordant sexual interactions individuals of a given relationship type and sexual activity stratum have over 250 days with no intervention (Panel A) or intervention only in the 25% of men most likely to have a one-time partner (Panel B). Rows indicate relationship type (preferred number of main (M) and casual (C) partners), while columns indicate sexual activity strata, or a node’s daily probability of having a one-time partner.

Given that universal behavior change is unlikely, we also examined a scenario with a targeted intervention, such that only men with the highest-risk sexual behavior (those in strata 5 and 6 of the daily probability of having a one-time partner) changed their behavior and received vaccination (Figure 4). In this simulation, we saw minimal impact on intervention efficacy (mean: 11.97% $P_{25\%}$ and $P_{75\%}$: 10.39%, 14.23%), indicating that the majority of prevented cases are due to behavior change by individuals most likely to form one-time partnerships. The effect of this targeted intervention can also be seen in infection risk, or the number of sexual interactions a node has as part of a serodiscordant partnership (Figure 5). While the number of at-risk sexual contacts does increase with a node’s degree, or number of partners, it is most affected by whether a node is in the highest-risk sexual behavior group (those in strata 6 of sexual activity, and to a lesser degree, strata 5). However, implementing behavior change decreases the average number of at-risk sexual contacts these nodes have over the course of the epidemic, lowering their risk of infection.

Sensitivity analyses examining reduced compliance to isolation from main and casual partnerships are available in the appendix.

### 3.2 Comparison of intervention timings

**Fig. 6:**
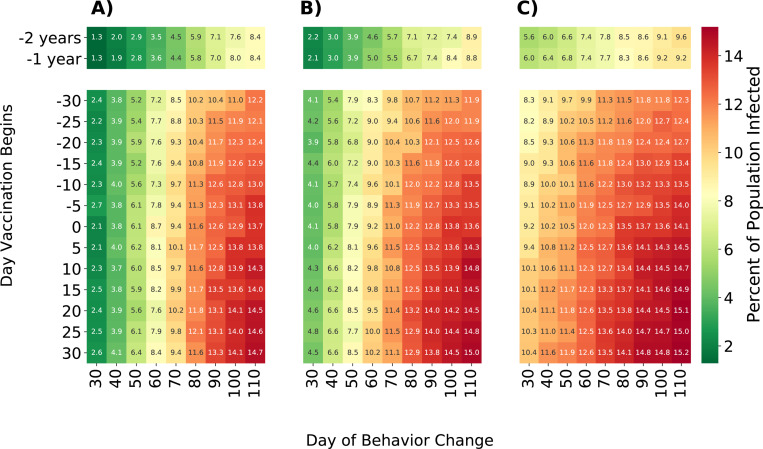
Percent of population infected with mpox after 250 days under different intervention timings and intensities. Interventions only affect men in strata 5 and 6 of sexual activity. Cell values indicate the percent of the network infected after 250 days. Rows indicate the day that vaccines become available; negative numbers indicate vaccination becoming available prior to the start of the outbreak. Columns show simulations where individuals reduce their probability of having a one-time partner by 75%, 50%, and 25%, respectively.

To better understand the impact of intervention timing, we compared the trade-off of the intensity of individual behavior change with the timing of the intervention. We compared three intensities of behavior change; reducing the probability of a one-time partner by 25%, 50%, or 75%, and several different intervention timings. Vaccination initiation ranged from 30 days prior to any outbreak to 30 days after the outbreak began and behavior change initiation ranged from 30 to 110 days after the outbreak began (Figure 6). As expected, earlier and more intense interventions result in fewer overall infections. Beginning vaccination a year before the outbreak results in only 5.5% of men being infected, averting 950 infections or nearly 10% of the total population in our model. However, it is evident that even for early initiation of a vaccination campaign, individual behavior modification has an important role, reducing the overall number of individuals infected from 15.2% to 1.3% of the network when comparing scenarios with the least intense and latest to start behavioral change to scenarios with the most intense and earliest behavior change.

### 3.3 Infection Dynamics

**Fig. 7:**
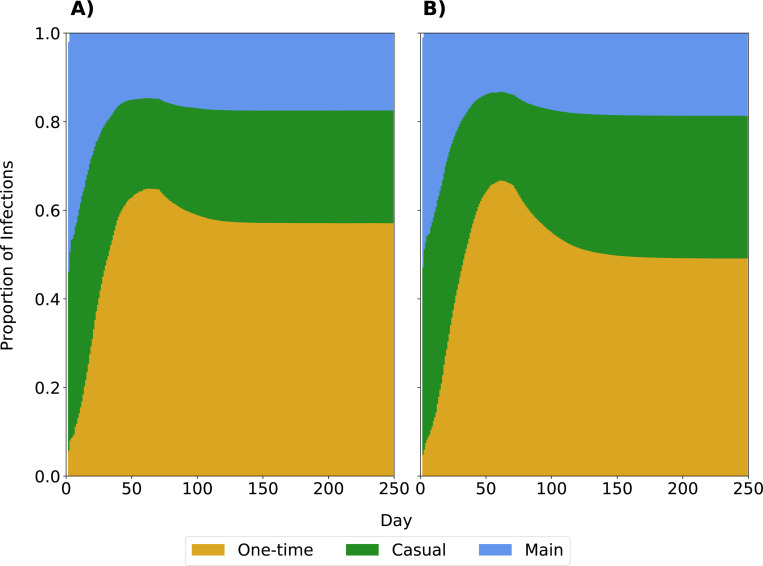
Proportion of infections attributable to each relationship type. Panels indicate the proportion of cumulative infections by time t attributable to each relationship type when intervention occurs only in the 25% of men most likely to have a one-time partner. Panel A shows the results with full isolation compliance after diagnosis; Panel B shows results with partial compliance.

We tracked the source node of every infection in our model, allowing us to calculate the proportion of infections attributable to each relationship type. Figure 7 shows the proportion of infections at time t attributable to each infection type with full isolation compliance (Panel A) and partial isolation compliance (Panel B) in the scenario with behavior change introduced on day 70 and vaccination at day 30. In either scenario, the early outbreak is driven by main and casual partnerships. Within 50 days, more than half of total infections are attributable to one-time partnerships. The relatively sharp decrease in the proportion of infections attributable to one-time partnerships is due to the introduction of behavior change on day 70, showcasing the impact of even 25% of individuals reducing their probability of having a one-time partnership. In the partial isolation compliance scenario, when individuals are isolated, they still have some possibility of interacting with their main or casual partners. Thus, a larger proportion of infections come from these relationships. There is greater between-simulation variability in these proportions earlier, given that early infections may be driven by whether the initially infected nodes have main and casual relationships as well as the variability to the smaller number of total infections early in the outbreak (Figure A4).

**Fig. 8:**
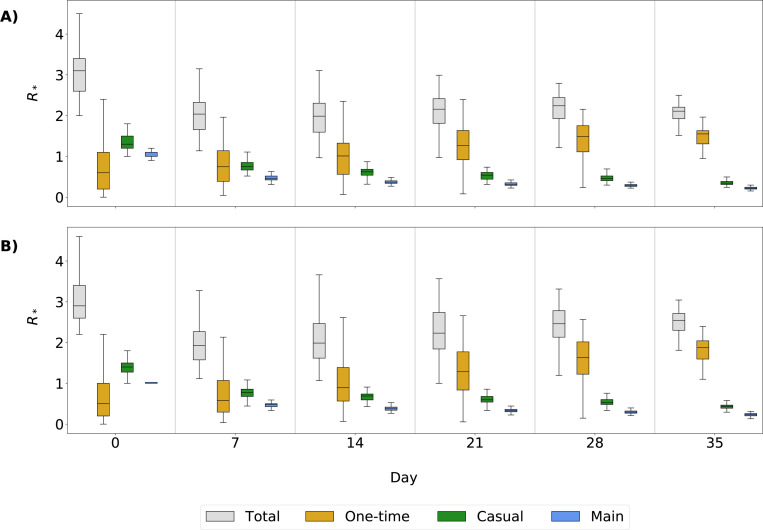
Effective reproductive number $\left(R_{*}^{t}\right)$ at time t. Box plots show the results of $R_{*}^{t}$ for main, casual, one-time, and total partnerships. Panel A shows results from simulations with full isolation compliance; Panel B shows results from simulations with partial isolation compliance. Time t=0 is a special case of $R_{*}^{t}$ which is equivalent to $R_{0}$.

This can also be seen in the $R_{*}^{t}$ values for each relationship type at different time steps (Figure 8). Values at time t=0 are equivalent to $R_{0}$, the average number of infections per initially infected node. The $R_{*}^{t}$ value for one-time partnerships is initially lower than the $R_{*}^{t}$ values for main and casual partners but becomes relatively more important to overall transmission within the first 14 days of the simulation. At t=0, median $R_{*}^{t}=1.30$ for casual partnerships ($P_{25\%}$ and $P_{75\%}$: 1.20, 1.50), 1.00 for main ($P_{25\%}$ and $P_{75\%}$: 1.00, 1.10), and 0.6 for one-time ($P_{25\%}$ and $P_{75\%}$: 0.20, 1.10). By t=28, the median $R_{*}^{t}$ for one-time partnerships has more than doubled to 1.48 ($P_{25\%}$ and $P_{75\%}$: 1.11, 1.75). At the same time, it decreased for casual and main partnerships: median $R_{*}^{t}=0.46$ ($P_{25\%}$ and $P_{75\%}$: 0.40, 0.52) and 0.29 ($P_{25\%}$ and $P_{75\%}$: 0.27, 0.31), respectively. This change in the $R_{*}^{t}$ values indicates that the continuation of the outbreak after this point is largely due to spread via one-time partnerships.

## 4 Discussion

This study integrated data about the clinical course of mpox, sexual network characteristics of MSM, and individual- and policy-level interventions to generate a mechanistic network model of mpox transmission in a US-based population of MSM. We describe simulations that compare the impact of the timing of interventions at different levels of compliance and intervention access.

We found that individual behavior change, reducing the probability of having a one-time partner, can reduce the number of individuals infected, even if implemented only among individuals with high-risk sexual behavior.The timing of interventions also played a crucial role in the final outcome of our modeling. We show that earlier implementation of behavior change and vaccination has a greater impact on reducing the spread of mpox. This is particularly true if behavior change begins in the early phases of an outbreak when vaccine coverage may be limited, suggesting that rapid deployment of public health messaging and vaccination campaigns at the onset of an outbreak is vital. However, even when vaccination was delayed, individual behavior modification mitigated transmission. This finding is particularly relevant in settings where vaccine supply may be limited or slow to roll out.

While intense and early behavior change can clearly reduce mpox transmission in our simulations, it can be burdensome and difficult to sustain. We present different combinations of intervention intensity and timing for behavior change with vaccination timing. Our results suggest that better epidemic preparedness, namely better vaccine coverage prior to the start of the epidemic, can offset variations in individual behavior change. Even in scenarios where only high-risk individuals were vaccinated, our study showed that beginning vaccination a year prior to the outbreak can be as effective at preventing transmission as behavior change. If intervening on a subset of the population with the highest risk leads to a sufficient decrease in cumulative infection, it could be possible to tailor public messaging and communication campaigns to this group.

Finally, our model contributes insight into the differential impact of relationship types on transmission dynamics over time. By tracking the source of each infection during modeling, we are able to present $R_{0}$ and $R_{*}^{t}$ values for each relationship type and at different times during the outbreak. Our results show that main and casual partnerships drive the early spread of infections, while one-time relationships connect the sparse network over time. These connections prevent the infection from remaining constrained to a limited portion of the network, particularly in scenarios where behavior change is delayed or limited. Additionally, we show that individuals who are most likely to form one-time partnerships have, on average, a greater number of at-risk (serodiscordant) sexual interactions than individuals with the same average number of partners but whose partners are sustained. These results are particular to the mpox outbreak in 2022, but could inform responses to future mpox outbreaks, or STIs more broadly, by underlining the importance of widespread vaccination as well as the impact of timely behavioral adaptations during STI outbreaks.

Our results showing the protective effect of behavior change and vaccination during the 2022 outbreak are consistent with those of studies using other modeling approaches, such as statistical network models[15, 17, 19]. We found a greater impact of behavior change alone than Clay et al., approximately 30% of cases were averted in our scenario after 250 days compared to 25% after 1 year. However, they found a greater impact of vaccination and behavior change together, averting 84% of cases after a year compared to 30% after 250 days in our results. The greater impact of vaccination in the modeling from Clay et al. could be due to the longer time frame, given that vaccine roll out was relatively slow. Additional sources of variation could be due to different assumptions about the level of behavioral change, vaccine efficacy, and the natural history of the disease.

Comparing disparate methods gives us greater confidence in common results, such as the efficacy of behavioral adaptation and vaccination in outbreak prevention. However, the mechanistic modeling approach allows us unique insight into this outbreak and potential future outbreaks of mpox that other modeling approaches cannot capture. First, we are able to incorporate individual-level action, such as heterogeneity in sexual behavior and changes in partnership seeking. We are also able to capture more realistic sexual behavior that other popular modeling approaches, such as compartmental models, are unable to model, namely, the importance of repeated sexual partnerships. Secondly, our model is able to track the source of each infection, so we are able to show the proportion of infections attributable to each relationship type in each simulation scenario, providing important information about the effect of behavior change and isolation on the dynamics of the outbreak. Finally, our model is readily interpretable and flexible. By modeling and intervening on the network at the level of the individual, translating modeling results to potential disease prevention recommendations, or updating the model with new information and exploring new interventions, is relatively straightforward.

Our study has several limitations. First, while this model parameterized the simulated sexual networks based on survey data from MSM in the US, this may not be generalizable to other populations of MSM[21, 23], and is not directly generalizable to a heterosexual population. The network parameter estimates we use are pulled from multiple studies, which could introduce additional uncertainty in the model. However, this approach is also taken in other modeling studies for mpox [15, 19] and has the benefit of allowing our results to be more easily compared because the network parameterization is more similar. Secondly, the assumptions about isolation and diagnosis delays were based on early outbreak data, which may not fully reflect evolving clinical practices and public awareness as the outbreak progressed. Future studies could explore the impact of varying these parameters to reflect different healthcare settings and policy environments that impact healthcare access. Future work could also extend this modeling strategy to include assortative partner selection by individuals’ demographics, such as age and race or ethnicity, and the potential impact of co-infection with other STIs, particularly HIV. Finally, we are limited by the lack of computationally scalable inferential techniques for mechanistic network models. Despite these limitations, our modeling provides valuable and unique insights.

## 5 Conclusions

Mechanistic network models provide a valuable framework for understanding the transmission dynamics of mpox in MSM populations. Our results suggest that early and sustained public health interventions, combining behavior change with vaccination, are critical to controlling the spread of mpox. Tailoring interventions to target high-risk groups, particularly those engaging in one-time partnerships, can optimize the use of limited resources and achieve meaningful reductions in transmission. These insights have important implications for public health strategies in the face of future outbreaks of mpox or similar STIs.

## Supplementary Material

Supplement 1

## Figures and Tables

**Table 1:** Parameters for simulation scenarios

**Network Model**		

$rt_{k}$		Relationship type, percent of nodes [21, 23]
	47.1	0 main, 0 casual
	16.7	0 main, 1 casual
	7.4	0 main, 2 casual
	22.0	1 main, 0 casual
	4.7	1 main, 1 casual
	2.1	1 main, 2 casual
$\pi_{o,k}$		Daily probability of one-time partnership formation [21, 23]
	0	stratum 1
	0.001	stratum 2
	0.0054	stratum 3
	0.0101	stratum 4
	0.0315	stratum 5
	0.286	stratum 6
$n_{o}$	Geometric($1-\pi_{o,k}$)	Number of one-time contacts, per day [21]
$rd_{m,e}$	Geometric(1/407)	Duration of main partnership, days [21, 23]
$rd_{c,e}$	Geometric(1/166)	Duration of casual partnership, days [21, 23]

**Epidemic Model**		

β	0.9	Probability of transmission per sexual contact
$\pi_{m}$	0.22	Daily probability of sexual contact, main partnership [21, 23]
$\pi_{c}$	0.14	Daily probability of sexual contact, casual partnership [21, 23]
$t_{e,k}$	Normal(7, 1)	Time spent in exposed state, days [1, 2, 13, 25–27]
$t_{i,k}$	Normal(27, 3)	Time spent in infectious state, days [1, 2, 13, 25–27]

**Interventions**		

$\pi_{b,k}$	0.5	Decrease in probability of one-time partnership formation
$\pi_{v_{1}}$	0.14	Probability of vaccination, only one dose [32]
$\pi_{v_{2}}$	0.227	Probability of vaccination, two doses [32]
$VE_{1}$	0.358	Vaccine efficacy, one dose [34]
$VE_{2}$	0.66	Vaccine efficacy, both doses [34]

Node-specific attributes have the subscript ‘$X_{x,k}$’, edge-specific attributes have the subscript ‘$X_{x,e}$

## Data Availability

All data and code used for this manuscript have been made publicly available at https://github.com/onnela-lab/mpox-model.

## Abbreviations

CDC: Centers for Disease Control and Prevention
HIV/AIDS: Human immunodeficiency virus/Acquired immunodeficiency syndrome
MSM: Gay, bisexual, and other men who have sex with men
STI: Sexually transmitted infection
